# Supplementary material for: An evolutionarily conserved metabolite inhibits biofilm formation in Escherichia coli K-12
Source: Nat Commun. 2024 Nov 21;15:10079. doi: 10.1038/s41467-024-54501-w (PMC11582573; doi:10.1038/s41467-024-54501-w)
Supplement: Supplementary file 1 — Supplementary Information [file 41467_2024_54501_MOESM1_ESM.pdf]

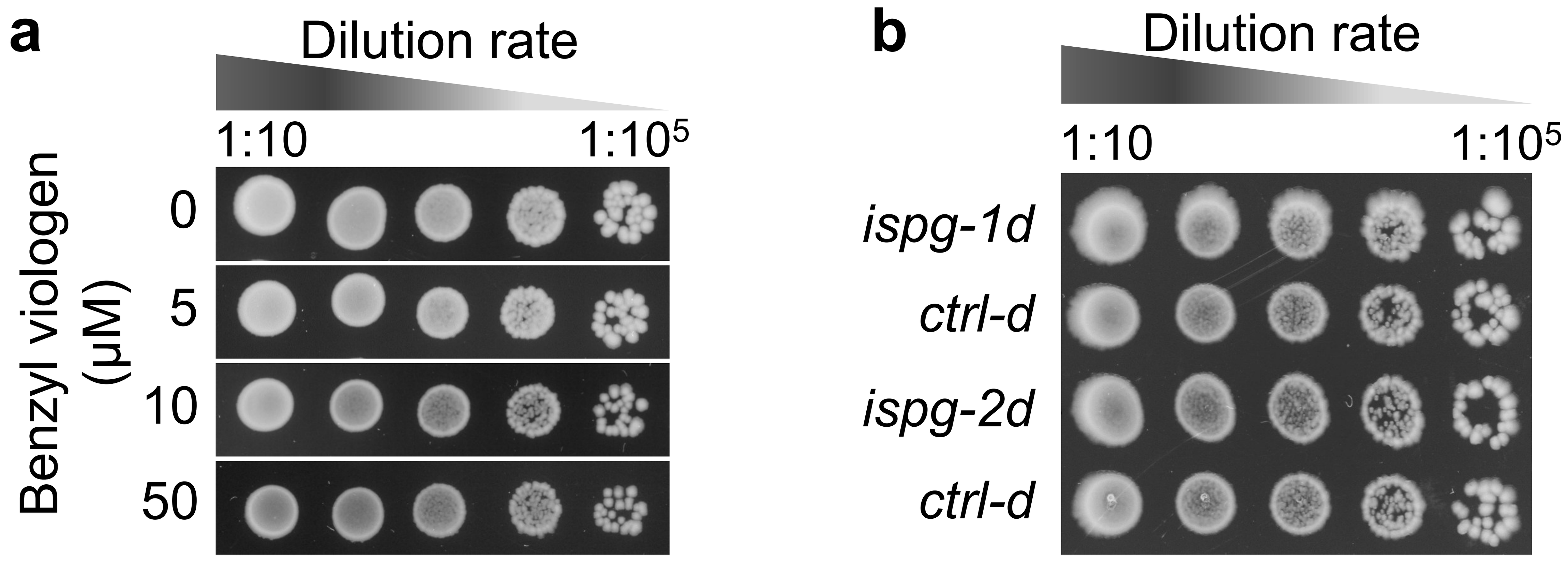

Supplementary Figure 1. Colony phenotypes of CRISPRi strains and benzyl viologen-treated *E. coli.*

Colony phenotype analysis of serially diluted cultures (in 10-fold steps, ranging from 1:10 to 1:100,000) of the MG1655 strain grown overnight on LB plates at 37°C. **a** Colony phenotypes observed on LB plain medium supplemented with increasing concentrations of benzyl viologen and **b** on LB plates supplemented with carbenicillin and chloramphenicol antibiotics for the CRISPRi strains (*ispg-1d*, *ispg-2d*, and *ctrl-d*). OD_{600nm} measured after a 1:10 dilution is 0.1 for **a** and 0.07 for **b**.

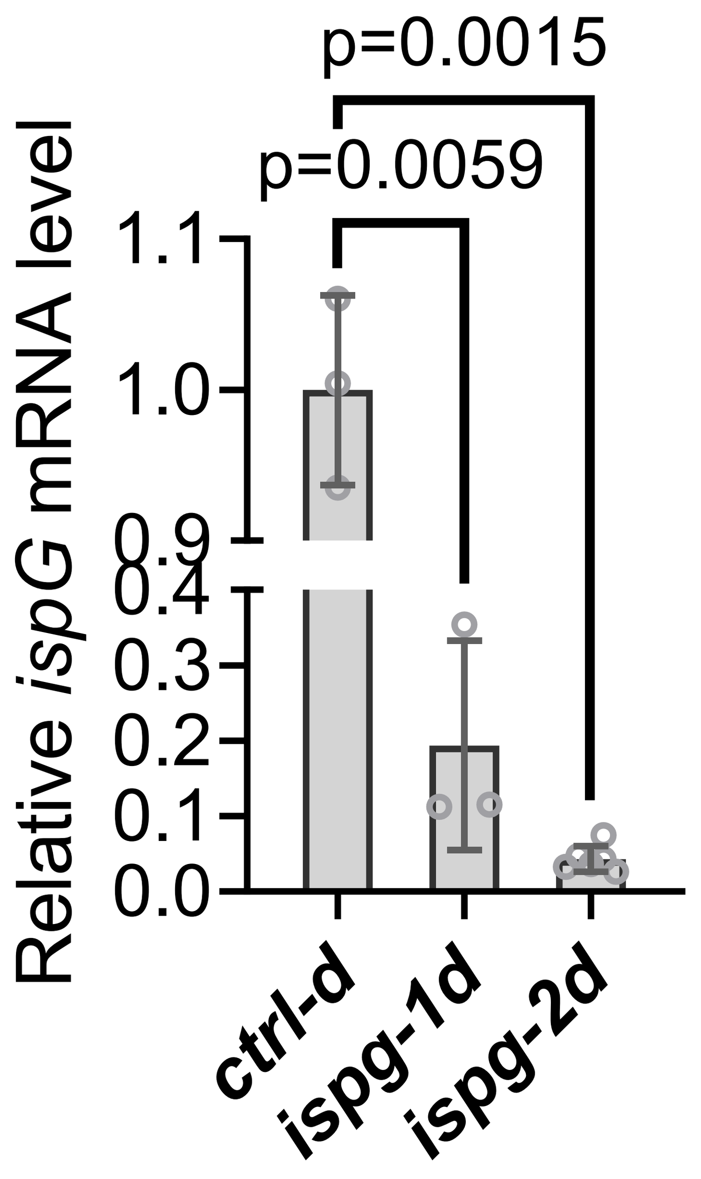

Supplementary Figure 2. Knockdown of *ispG* expression in two independent CRISPRi strains.

Relative expression levels of *ispG* in CRISPRi strains are assessed using RT-qPCR. Bars represent mean value and error bars show the standard deviation. Statistical analyses used are Brown-Forsythe and Welch ANOVA and two-sided Dunnett's multiple comparisons tests. Source data are provided in a Source Data file.

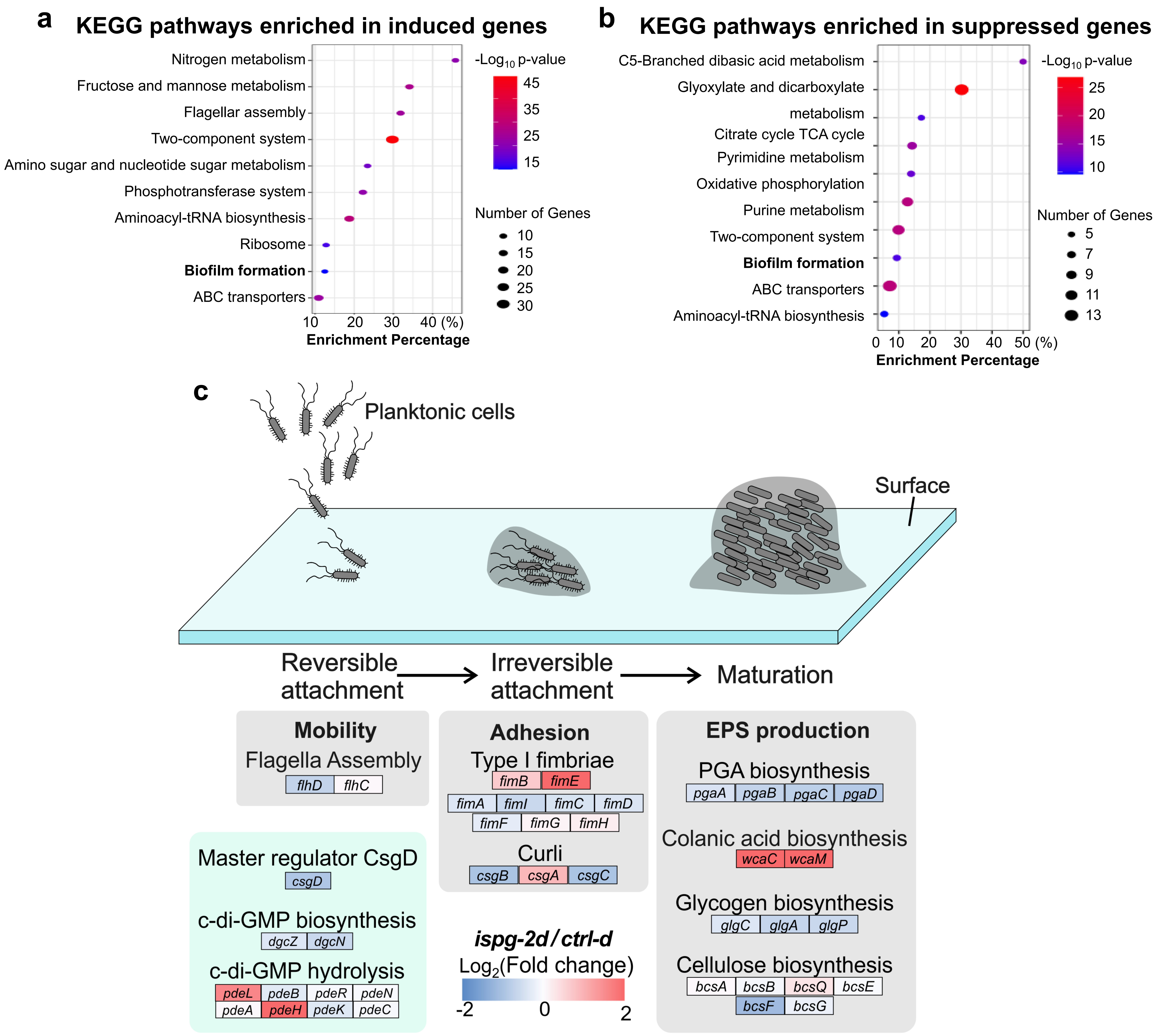

Supplementary Figure 3. Changes in transcript levels associated with biofilm formation in *E. coli* with elevated MEcPP accumulation.

a, b KEGG pathway enrichment analysis for induced (**a**) and suppressed (**b**) genes. Each bubble represents a specific KEGG pathway, with the size of the bubble corresponding to the number of differentially expressed genes (DEGs) associated with that pathway. The enrichment percentage for each pathway was calculated as: Enrichment Percentage = 100×(Number of DEGs /Total number of genes in the pathway). Pathways are ranked by their enrichment percentage. The color key reflects the $-\text{Log}_{10}$ of the p-value. **c** Schematic representation of expression changes in biofilm-related genes. The upper diagram outlines three key developmental stages of biofilm formation. Lower panels illustrate the expression patterns of regulatory or structural genes critical for biofilm formation at each stage. Genes within boxes shaded blue or red indicate down- or up-regulation, respectively, in the *ispg-2d* strain compared to the control (*ctrl-d*). The scale bar represents the $\log_2$ fold changes in expression levels.

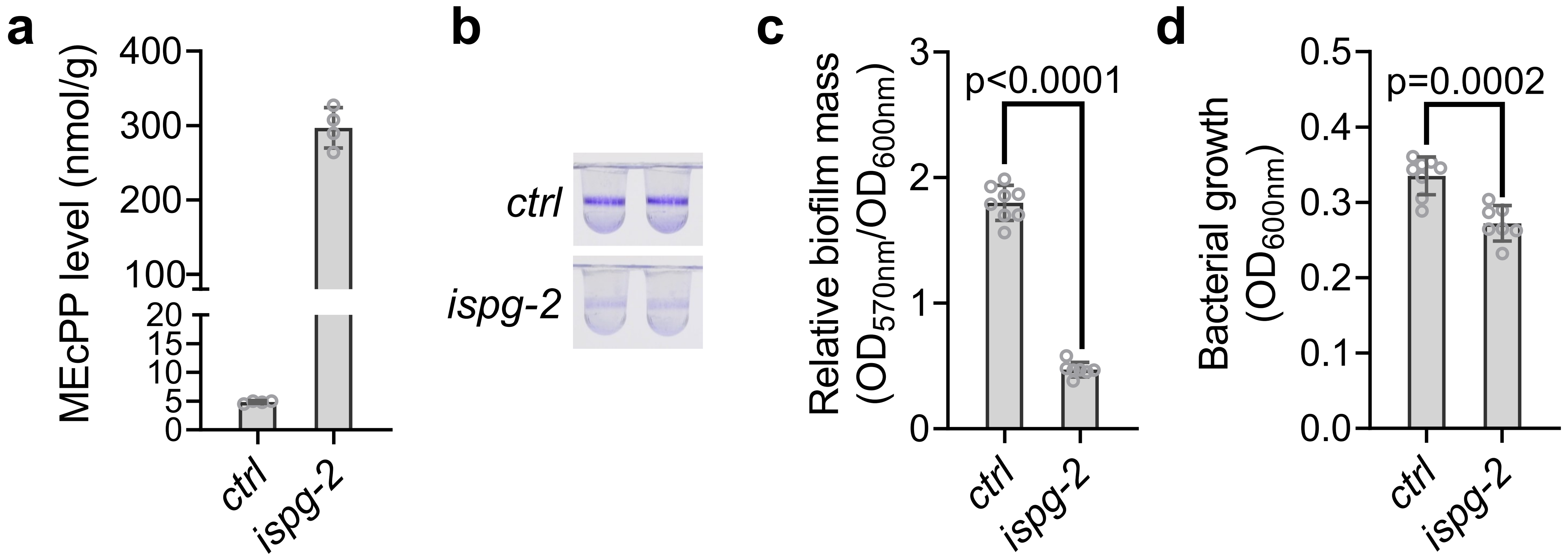

Supplementary Figure 4. Merged 2-in-1 CRISPRi *ispg-2* construct accumulate MEcPP and suppress biofilm formation.

a MEcPP level in the merged 2-in-1 CRISPRi *ispg-2* and *ctrl* strain. **b** crystal violate-visualized biofilm produced by *ispg-2* and *ctrl* strain statically grown at room temperature. **c, d** Relative biofilm mass calculated by the ratio between the OD_{570nm} of dissolved crystal violet-stained biofilm and the OD_{600nm} of planktonic cells at the time of staining (**c**), and bacterial growth measured at OD_{600nm} (**d**) for both strains. Bars represent mean value and error bars show the standard deviation. The statistical tests used are unpaired t-tests with Welch's correction (two-tailed). Source data are provided in a Source Data file.

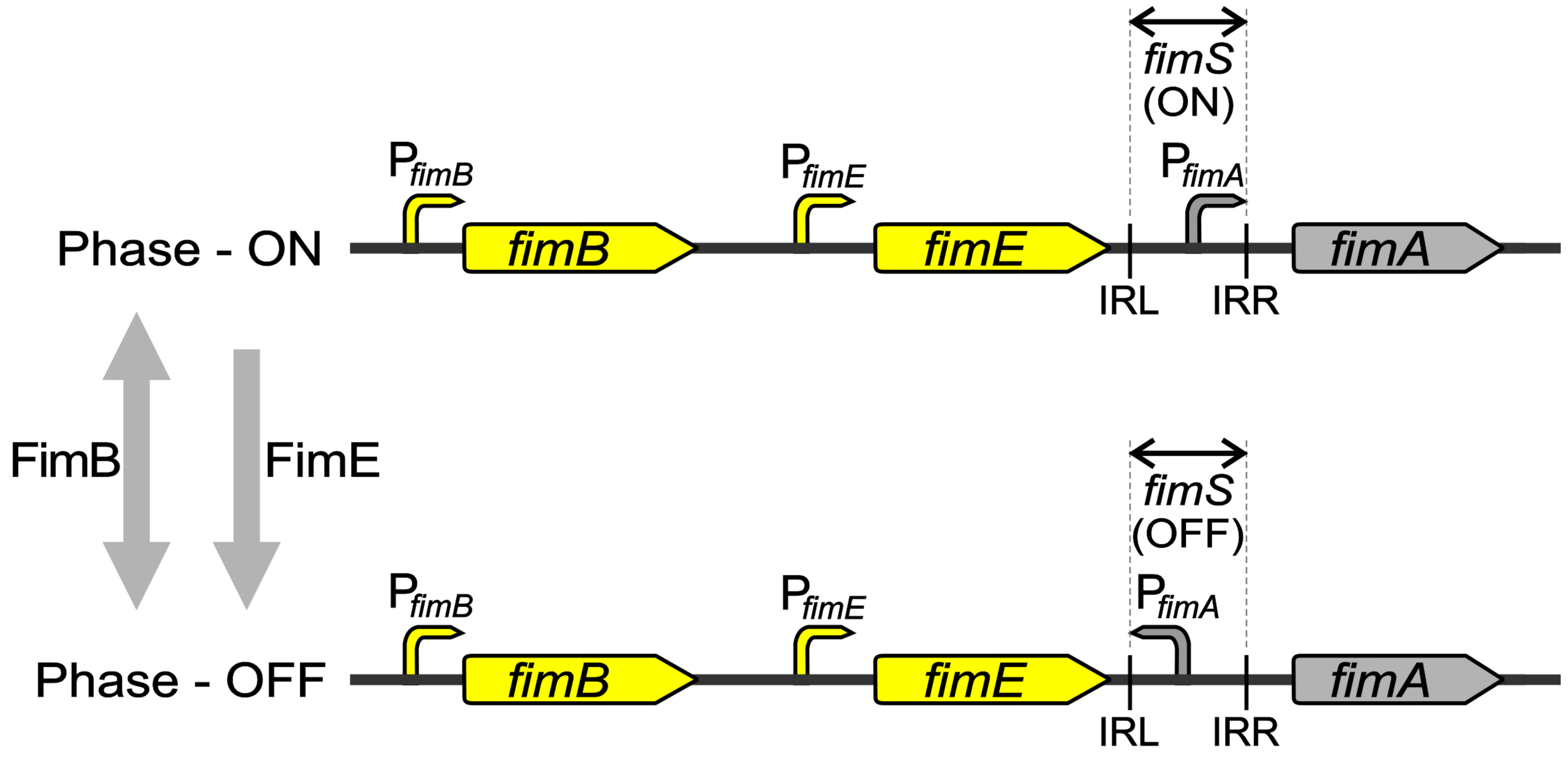

Supplementary Figure 5. Diagram of the phase variations in *fim* operon.
The diagram shows the phase variations of *fimA* promoter within the *fim* operon. Two site-specific recombinases, FimB and FimE, bind to inverted repeat left (IRL) and inverted repeat right (IRR) mediating the inversion of the *fimS* element between the ON and OFF phases. This inversion controls the orientation of the *fimA* promoter. FimE primarily facilitates the transition from the ON to OFF phase, leading to reduced fimbriae production. In contrast, FimB can switch *fimA* promoter in both directions, thereby maintaining a dynamic balance in fimbrial expression.

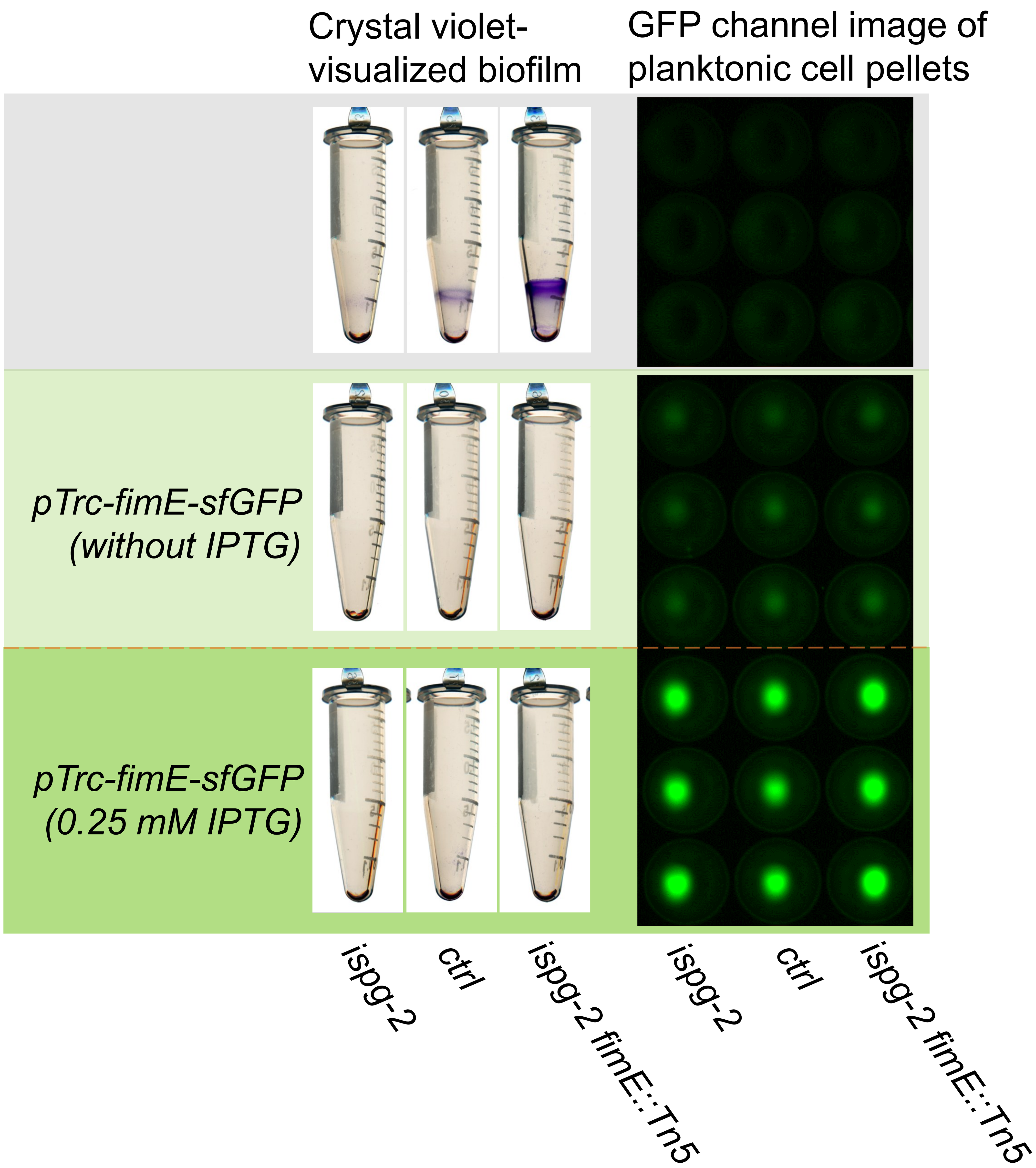

Supplementary Figure 6. Overexpression of *fimE* abolish biofilm.
Across three strains of *E. coli* (*ispg-2*, *ctrl*, and *ispg-2 fimE::Tn5*), varying levels of biofilm production are observed in the top row. Both leaky expression and IPTG-induced expression of *Ptrc*-driven *fimE-sfGFP* (middle and lower rows, respectively) result in the abolition of biofilm formation across all three genotypes. In the middle column, biofilm formed in polypropylene tubes is visualized via crystal violet staining. The right column displays the expression of *FimE-sfGFP* in different strains, with or without IPTG induction, by imaging the pellets of planktonic cells in the GFP channel.

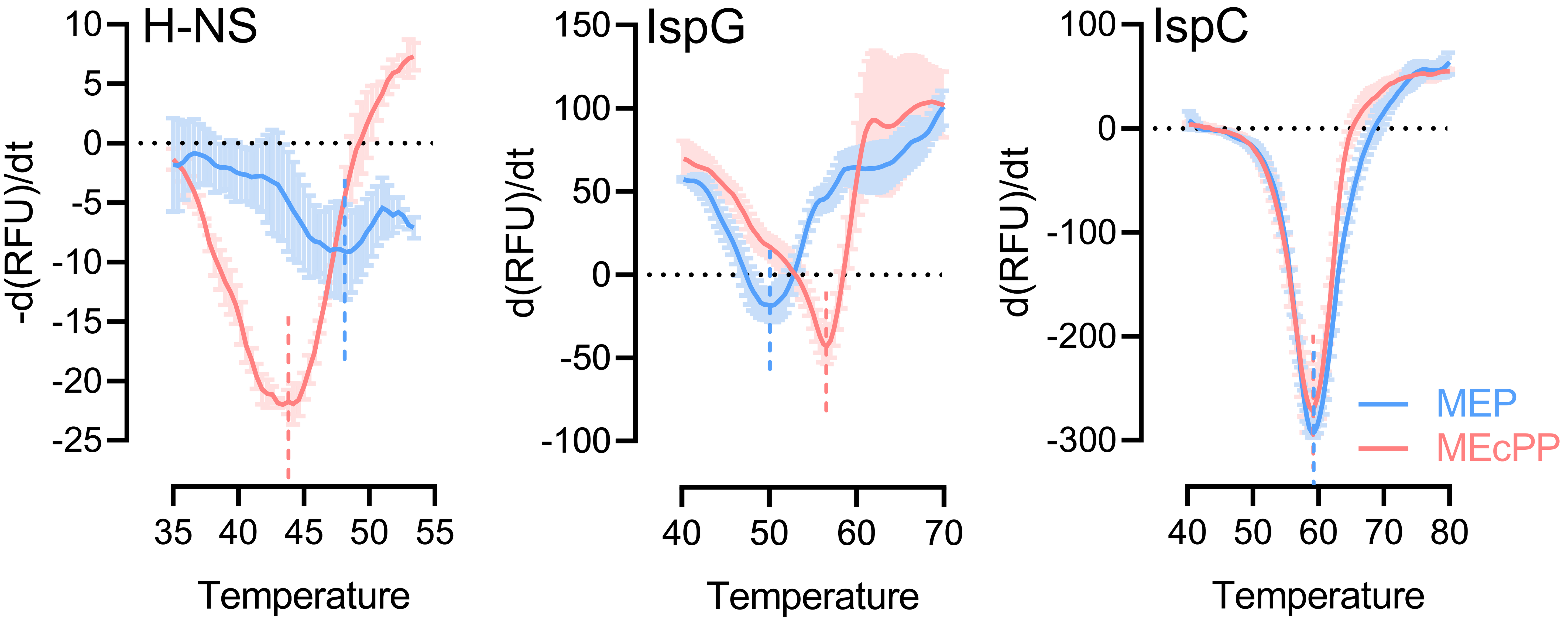

Supplementary Figure 7. Interaction between MEcPP and H-NS using protein thermal shift assay.

Protein thermal shift assay demonstrating a shift in the melting temperature of H-NS in the presence of MEcPP, but not its analog MEP. IspC and IspG serve as negative and positive controls, respectively. Data are presented as mean values +/- SD. Source data are provided in a Source Data file.

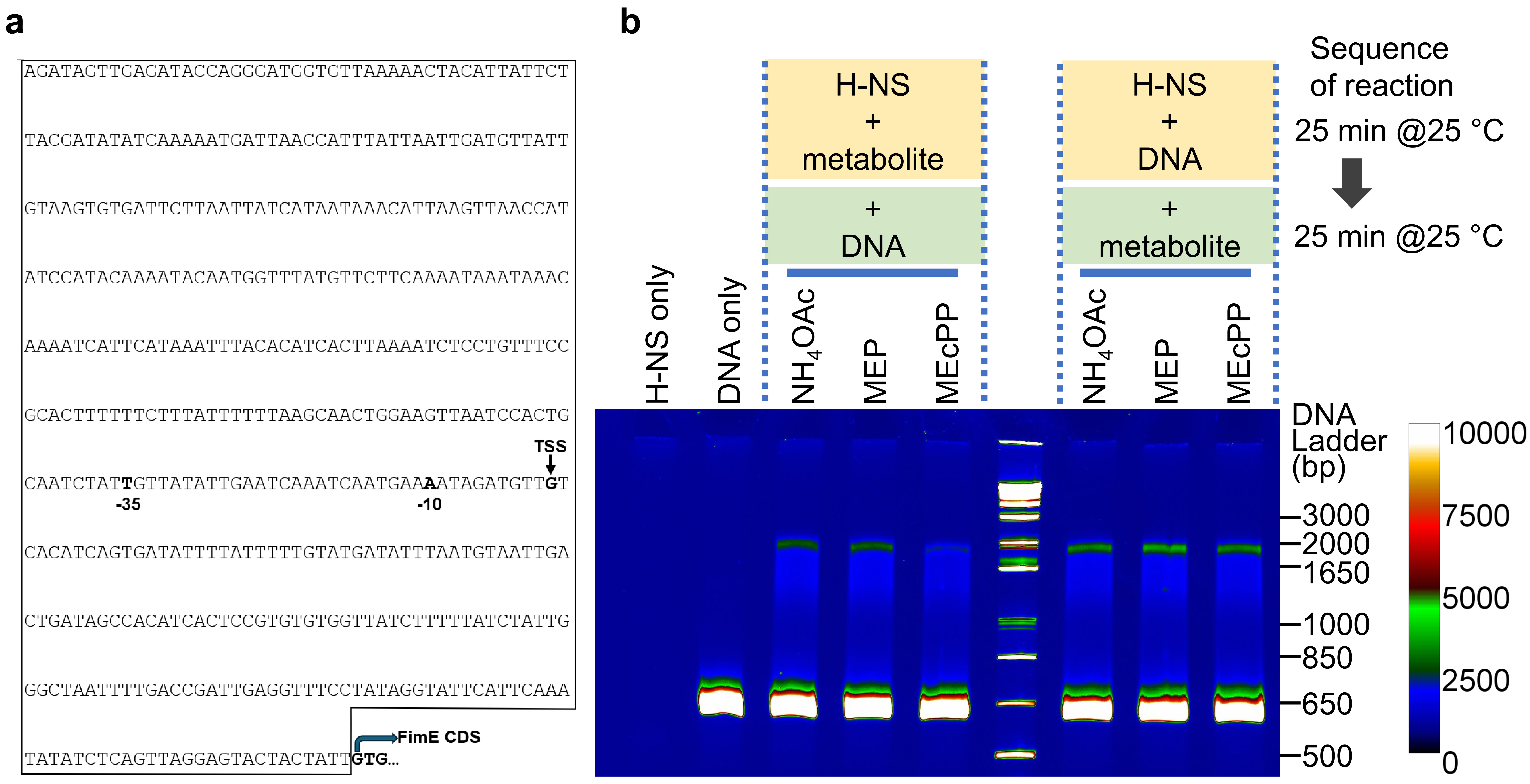

Supplementary Figure 8. Preincubation of H-NS with MEcPP reduces mobility shift of *fimE* promoter.

a. Nucleotides sequence of the PCR-amplified *fimE* promoter fragment (boxed text) with the transcriptional start site (TSS), -10, and -35 boxes indicated. **b**. Electrophoretic Mobility Shift Assay (EMSA) shows a reduction in the pool of shifted *fimE* promoter DNA fragments when H-NS is preincubated with 200 μM MEcPP, but not with the same concentration of its analog MEP or NH_4OAc. Change of the mobility shift induced by MEcPP treatment was not observed when H-NS is already complexed with *fimE* promoter DNA fragments. Similar results were observed in two additional experiments. Source data are provided in a Source Data file.

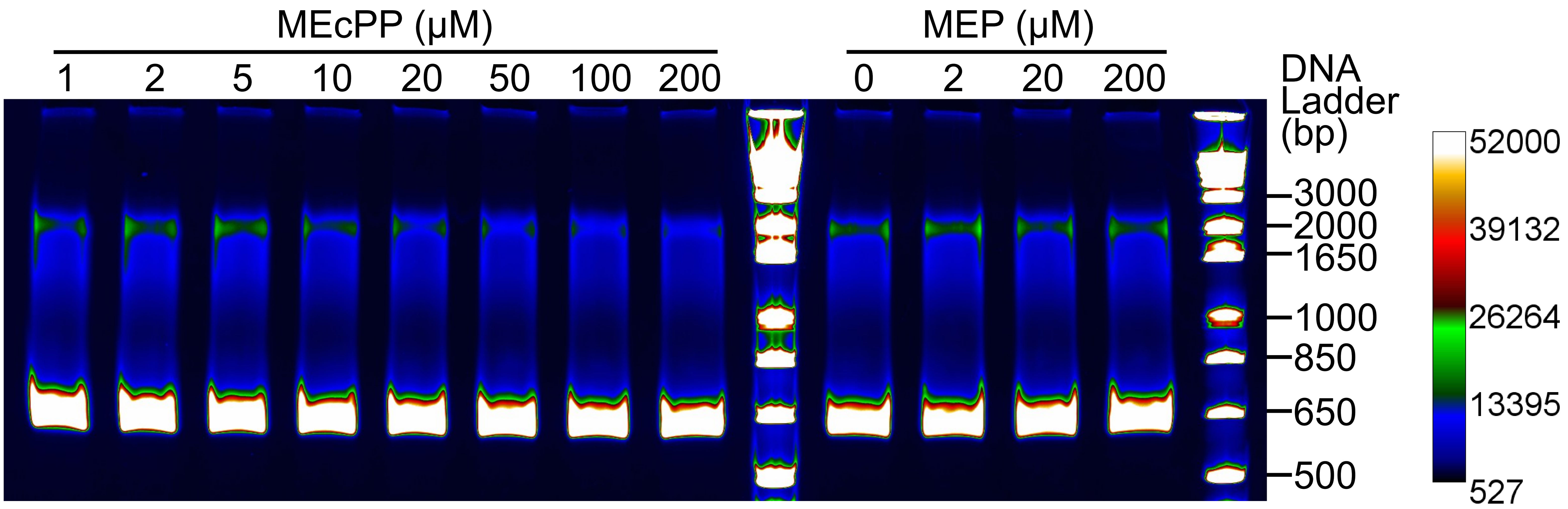

Supplementary Figure 9. MEcPP reduces association between H-NS and *fimE* promoter. Electrophoretic Mobility Shift Assay (EMSA) shows that increasing concentrations of MEcPP, but not its analog MEP, lead to a reduction in the pool of shifted *fimE* promoter DNA fragments. Similar results were observed in two additional experiments. Source data are provided in a Source Data file.

Supplementary Table 1. List of plasmids (sources and construction details)

Plasmid	Plasmid base	Reference /Source	Primers	Construction strategy
pgRNA-bacteria	pUC19	Gift from Stanley Qi (Addgene plasmid # 44251)[1]	N/A	N/A
pdCas9-bacteria	p15A	Gift from Stanley Qi (Addgene plasmid # 44249)[1]	N/A	N/A
pSNC-mTn5	pMMB208	Gift from Huatao Guo[2]	N/A	N/A
pSIJ8	pKD46	Gift from Alex Nielsen (Addgene plasmid # 68122)[3]	N/A	N/A
pET16b-IspC	pET-16b	[4]	N/A	N/A
pJB005	pTrcHisB	Gift from Keith Tyo (Addgene plasmid # 65468)[5]	N/A	N/A
pgRNA-ispg-1	pgRNA-bacteria	This study	NT2_Ec_F gtcatggactgtacggcgatGTTTTAGAGCTAGAAATAGCAAGTTAAAATAAGGC<br><br>Ec_R ACTAGTATTATACCTAGGACTGAGCTAGC	PCR amplify the plasmid base pgRNA-bacteria with the primer pair, then self-ligate into a complete plasmid. Primer NT2_Ec_F has an anchor of the sgRNA sequence (lowercase letter) targeting site 1 on the non-template strand of *ispG*.
pgRNA-ispg-2	pgRNA-bacteria	This study	NT3_Ec_F gcttctgccgcgtccatcgtGTTTTAGAGCTAGAAATAGCAAGTTAAAATAAGGC<br><br>Ec_R ACTAGTATTATACCTAGGACTGAGCTAGC	PCR amplify the plasmid base pgRNA-bacteria with the primer pair, then self-ligate into a complete plasmid. Primer NT3_Ec_F has an anchor of the sgRNA sequence (lowercase letter) targeting site 2 on the non-template strand of *ispG*.

pgRNA-ctrl	pgRNA-bacteria	This study	mutNT3_Ec_F<br>gcttctgccgcgtccatccaGTTTTAGAGCTAGAAATAGCAAGTTAAAATAAGGC<br><br>Ec_R<br>ACTAGTATTATACCTAGGACTGAGCTAGC	PCR amplify the plasmid base pgRNA-bacteria with the primer pair, then self-ligate into a complete plasmid. Primer mutNT3_Ec_F has an anchor of the sgRNA sequence (lowercase letter) identical to sgRNA targeting site 2 on the non-template strand of *ispG*, except that two nucleotides immediate adjacent to the protospacer adjacent motif (PAM) site are substituted (gt to ca).
pgRNA-dCas9-ispg-2	pgRNA-ispg-2 and pdCas9-bacterial	This study	dCas9_fwd<br>ggctacgctcttca**CTT**AAGACCCACTTTCACATTTAAGTTG<br>dCas9_rev<br>ggctacgctcttct**ACC**gcggccgcgTAGCGAGTCAGTGAGCGAG<br><br>sgRNA_fwd<br>ggctacgctcttcc**GGT**cctgcaggtAATACGGTTATCCACagaatcag<br>sgRNA_rev<br>ggctacgctcttca**AAG**acgtcCTAGACTCGAGTAAGGATCcagttc	Golden Gate Assembly was used to merge the sgRNA and dCas9 plasmids into the backbone of the pgRNA-bacteria plasmid. Primer pair sgRNA_fwd and _rev was used to amplify the pgRNA-ispg-2 and pgRNA-ctrl, primer pair dCas9_fwd and _rev was used to amplify the dCas9 expression cassette. All amplified fragments were digested with SapI (recognition site underscored) to generate compatible sticky end (highlighted by bold uppercase letters), followed by ligation of the two fragments with T4 DNA ligase.
pgRNA-dCas9-ctrl	pgRNA-ctrl and pdCas9-bacterial	This study	dCas9_fwd<br>ggctacgctcttca**CTT**AAGACCCACTTTCACATTTAAGTTG<br>dCas9_rev<br>ggctacgctcttct**ACC**gcggccgcgTAGCGAGTCAGTGAGCGAG<br><br>sgRNA_fwd<br>ggctacgctcttcc**GGT**cctgcaggtAATACGGTTATCCACagaatcag<br>sgRNA_rev<br>ggctacgctcttca**AAG**acgtcCTAGACTCGAGTAAGGATCcagttc	
pET28a-H-NS	pET28a	This study	HNS_NdeI_Fw<br>aatCATATGagcgaagcacttaaaattctga<br>HNS_XhoI_Rv<br>aacaCTCGAGttattgcttgatcaggaaatcgtcg	The coding sequence of *hns* was amplified with primer pair HNS_NdeI_fwd and HNS_XhoI_rev. The purified PCR product and pET28a plasmid were digested with XhoI and NdeI, the resulted two DNA fragments were joined together by T4 DNA ligase.

pET28a-IspG	pET28a	This study	IspG_fwd<br>ctggtgccgcgcggcagccatatgcataaccaggctccaattc<br>IspG_rev<br>agtggtggtggtggtggtgcttatttttcaacctgctgaac	Plasmid pET28a was first linearized by double digestion with XhoI and NdeI, then the coding sequence of *ispG* was amplified with primer pair IspG_fwd and _rev. The two DNA fragments were assembled with NEBuilder HiFi assembly master mix.
pSIJ8-GmR	pSIJ8	This study	pSIJ8_GmR_Fw<br>gacagttaccaatgcttaatcagtgaTTGCCGTAGAAGAACAGCAAGG<br>pSIJ8_GmR_Rv<br>ttcaaatatgtatccgctcatgagacGGATTCAGTCGTCACTCATGGT	The purpose of this modification is to change Amp resistance to Gentamycin resistance for plasmid compatibility. Plasmid pSIJ8 was first linearized by double digestion with XmnI and BsaI-HFv2 (cuts in the AmpR coding region). Then the primer pair pSIJ8_GmR_Fw and Rv were used to amplify Gentamycin resistance cassette from plasmid pDONR207. Lastly, the linearized pSIJ8 plasmid was mixed with the above amplified Gentamycin resistance cassette with equal molar ratio and assembled with NEBuilder HiFi assembly master mix.
pJB005-GmR	pJB005<br><br>(pTrc plasmid expressing GFP in backbone of pTrcHisB)	This study	pSIJ8_GmR_Fw<br>gacagttaccaatgcttaatcagtgaTTGCCGTAGAAGAACAGCAAGG<br>pSIJ8_GmR_Rv<br>ttcaaatatgtatccgctcatgagacGGATTCAGTCGTCACTCATGGT	The purpose of this modification is to change Amp resistance to Gentamycin resistance for plasmid compatibility. Plasmid pJB005 was first linearized by double digestion with XmnI and BsaI-HFv2 (cuts in the AmpR coding region). Then the primer pair pSIJ8_GmR_Fw and Rv were used to amplify Gentamycin resistance cassette from plasmid pDONR207. Lastly, the linearized pJB005 plasmid was mixed with the above amplified Gentamycin resistance cassette with equal molar ratio and assembled with NEBuilder HiFi assembly master mix.

pTrc-fimE-FLAG	pJB005-GmR	This study	FimEflag_FimE_Fw<br>aaataaggaggaataaaccatgtgagtaaacgtcgttatcttac<br>FimEflag_FimE_Rv<br>atatggtaccagctgcagatctcgagaacctcttctctttttaatttttc<br><br>FimE_Flag_oligo_Fw<br>gaaaaattaaaaagagaagaggttggtggttctggtggttctgactacaaagatgacgatgacaagtga<br>FimE_Flag_oligo_Rv<br>tctgtactagttctagaaagctttcacttgtcatcgtcatctttgtagtcagaaccacca	Plasmid pJB005-GmR was digested with BamHI-HF and XhoI to remove avGFP downstream of the P_{trc}. The coding sequence of *fimE* were amplified with primer pair FimEflag_FimE_Fw/Rv. The two DNA fragments were then assembled into pTrc-fimE using NEBuilder HiFi assembly master mix. Plasmid pTrc-fimE was then digested with XhoI and EcoRI and is ready for insertion of the FLAG tag. To do so, two oligos containing FLAG tag coding sequence were first annealed into double strand (with 5' overhang on both ends), then were assembled into the digested pTrc-fimE plasmid using NEBuilder HiFi assembly master mix producing pTrc-fimE-FLAG.
pTrc-fimE-sfGFP	pJB005-GmR	This study	FimEsfGFP-FimE_Fw<br>aaataaggaggaataaaccatgtgagtaaacgtcgttatcttac<br>FimEsfGFP-FimE_Rv<br>Agaaccaccaccagaaccaccaccaacctcttctctttttaatttttc<br><br>FimEsfGFP-sfGFP_Fw<br>ggtggtggttctggtggtggttctatggtgagcaagggcgaggag<br>FimEsfGFP-sfGFP_Rv<br>catatggtaccagctgcagatttacttgtacagctcgtccatgcc	Plasmid pJB005-GmR was digested with BamHI-HF and XhoI to remove avGFP downstream of the P_{trc}. The coding sequence of *fimE* and *sfGFP* were amplified with primer pairs FimEsfGFP-FimE_Fw/Rv and FimEsfGFP-sfGFP_Fw/Rv respectively. All three purified fragments were then assembled using NEBuilder HiFi assembly master mix.

Supplementary Table 2. List of primers

Primer	Sequence	Purpose
Tn5Km1<br>BDC1	gtattactgtttatgtaagcagacag<br>ggccacgcgtcgactagtacNNNNNNNNNNacgcc	First round arbitrarily primed PCR for amplification of transposon flanking sequence
Tn5Km2<br>BDC2	cttgtgcaatgtaacatcagag<br>ggccacgcgtcgactagtac	Second round arbitrarily primed PCR for amplification of transposon flanking sequence
FimE_RC1A5_LP<br>FimE_RC1A5_RP	cacatcactccgtgtgtggt<br>ggacagtttggccccaattg	Genotyping primer for *fimE::Tn5*
FimE_HR_Fw<br>FimE_HR_Rv	tttcctataggtattcattcaaatatatctcagttaggagtactactattCTCTGGCCCGTGTCTCAAAA<br>tgtcttgtatttatttgttttttaactttattatcaataagttaaatcaTTTCTACGGGGTCTGACGCT	Primer pair for amplify *kan* to produce DNA fragment for knockout *fimE* through homologous recombination. Nucleotides in uppercase letter for annealing to *kan* sequence, while the nucleotides in lowercase is annealing to border sequence of *fimE* coding region.
FimS_HinfI_F<br>FimS_HinfI_R	agtgaacggtcccaccattaac<br>gccggattatgggaaagaaataatct	PCR amplification of *fimA* promoter. To be followed with Hinf I digestion to digtinguish ON and OFF state of *fimA* promoter
FimEPro_F<br>FimEPro_R	agatagttgagataccagggat<br>aatagtagtactcctaactgagat	PCR amplification of *fimE* promoter for EMSA assay
FimE_QPCR_F<br>FimE_QPCR_R	ttctaccgttcacccgttacg<br>aatatagcgtcagtccggtcag	QPCR primers for *fimE* (102 bp)
dnaG_QPCR_F<br>dnaG_QPCR_R	gctttgcgattggttttgcg<br>catccccgcatcaatcaatgac	QPCR primers for *dnaG* (103 bp)
secA_QPCR_F<br>secA_QPCR_R	tcatgctgcaaacgcttgac<br>tcgctgcaaacatggagaac	QPCR primers for *secA* (79 bp)
era_QPCR_F<br>era_QPCR_R	acgattgccgcaatagtgtc<br>acaaagtggacaacgtgcag	QPCR primers for *era* (136 bp)
gyrB_QPCR_F<br>gyrB_QPCR_R	ttcaacgaacgccttgatgc<br>aattctggcgaaacgtctgc	QPCR primers for *gyrB* (127 bp)
idnT_QPCR_F<br>idnT_QPCR_R	tgcaatacggcgttaatggc<br>ttggtgaaacgttgcgtacc	QPCR primers for *idnT* (91 bp)
hcaT_QPCR_F<br>hcaT_QPCR_R	gtgccgcaatgcagaatttg<br>tgttgatctcggcgatttgc	QPCR primers for *hcaT* (100 bp)
cysG_QPCR_F<br>cysG_QPCR_R	gaagcgcaaaaaggcaaacg<br>ttgcacagtgtttccagctc	QPCR primers for *cysG* (92 bp)
ispG_QPCR_F<br>ispG_QPCR_R	tacacggacgatatcagcgc<br>ttacgttgggaatgtgccga	QPCR primers for *ispG* (139 bp)

Supplementary References:

1. Qi LS*, et al.* Repurposing CRISPR as an RNA-guided platform for sequence-specific control of gene expression. *Cell* **152**, 1173-1183 (2013).

2. Naorem SS*, et al.* Efficient transposon mutagenesis mediated by an IPTG-controlled conditional suicide plasmid. *BMC Microbiol* **18**, 158 (2018).

3. Jensen SI, Lennen RM, Herrgard MJ, Nielsen AT. Seven gene deletions in seven days: Fast generation of Escherichia coli strains tolerant to acetate and osmotic stress. *Sci Rep* **5**, 17874 (2015).

4. Wang JZ*, et al.* Uncovering the functional residues of Arabidopsis isoprenoid biosynthesis enzyme HDS. *Proc Natl Acad Sci U S A* **117**, 355-361 (2020).

5. Sekar K, Gentile AM, Bostick JW, Tyo KE. N-Terminal-Based Targeted, Inducible Protein Degradation in Escherichia coli. *PLoS One* **11**, e0149746 (2016).
