# Supplementary material for: An evolutionarily conserved metabolite inhibits biofilm formation in Escherichia coli K-12
Source: Nat Commun. 2024 Nov 21;15:10079. doi: 10.1038/s41467-024-54501-w (PMC11582573; doi:10.1038/s41467-024-54501-w)
Supplement: Supplementary file 2 — Description of Additional Supplementary Files [file 41467_2024_54501_MOESM2_ESM.pdf]

Description of Additional Supplementary Files:

Supplementary Data 1: Targeted metabolomic analyses focusing on central carbon and energy metabolism (CCEM) in the CRISPRi strain *ispg-2d* and *ctrl-d*

Supplementary Data 2: Targeted CCEM metabolomics analysis method parameters

Supplementary Data 3: RNA-seq analyses of differentially expressed genes between *ispg-2d* and *ctrl-d* strains

Supplementary Data 4: List of differentially represented peptide features in the high-MEcPP *ispg-2d* and control *ctrl-d* strains in LiP-MS analysis

Supplementary Data 5: List of differentially represented peptide features in wild-type E. coli total protein treated with MEcPP or MEP in LiP-MS analysis

Supplementary Data 6: MS/MS spectrums and peak profiles of H-NS peptides

Supplementary Data 7: Quality control data and plots for the samples with limited proteolysis treatment
