# Supplementary material for: An evolutionarily conserved metabolite inhibits biofilm formation in Escherichia coli K-12
Source: Nat Commun. 2024 Nov 21;15:10079. doi: 10.1038/s41467-024-54501-w (PMC11582573; doi:10.1038/s41467-024-54501-w)
Supplement: Supplementary file 8 — Supplementary Data 6 [file 41467_2024_54501_MOESM8_ESM.pdf]

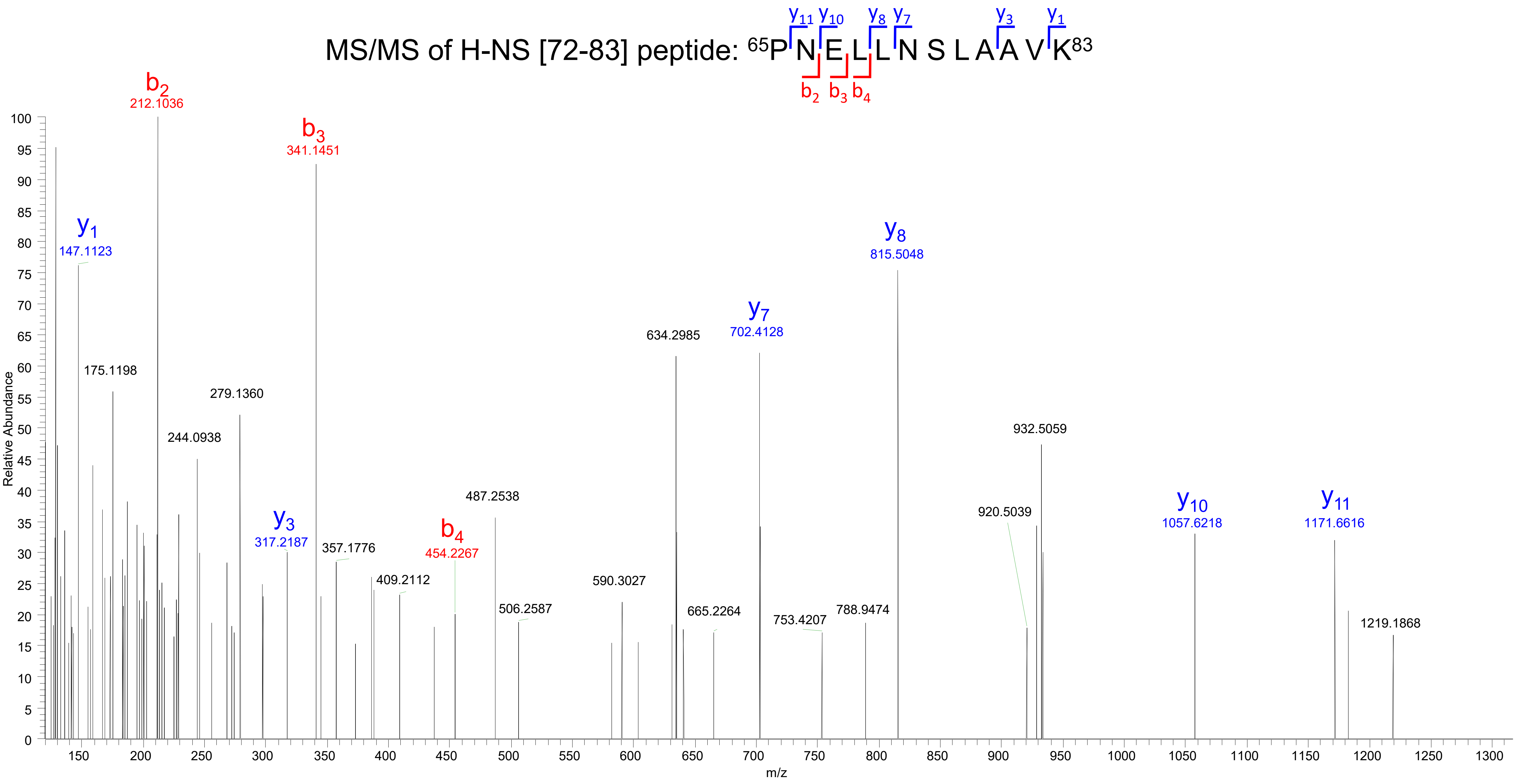

MS/MS of H-NS [72-83] peptide: 65P N E L L N S L A A V K83
y11 y10 y8 y7 y3 y1
b2 b3 b4
b2
212.1036
b3
341.1451
y1
147.1123
175.1198
279.1360
244.0938
y3
317.2187
357.1776
409.2112
b4
454.2267
487.2538
506.2587
590.3027
634.2985
665.2264
y7
702.4128
753.4207
788.9474
y8
815.5048
920.5039
932.5059
y10
1057.6218
y11
1171.6616
1219.1868
Relative Abundance
m/z

Extracted ion chromatography of HNS[72-83] peptide

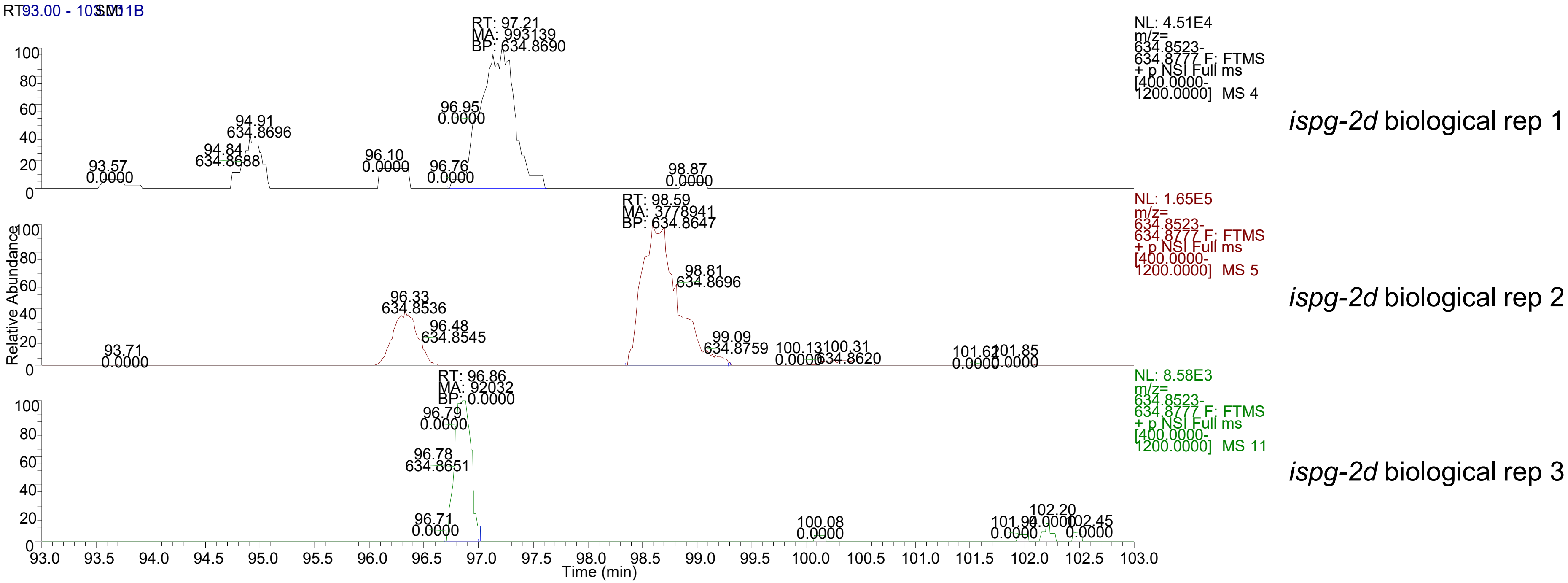

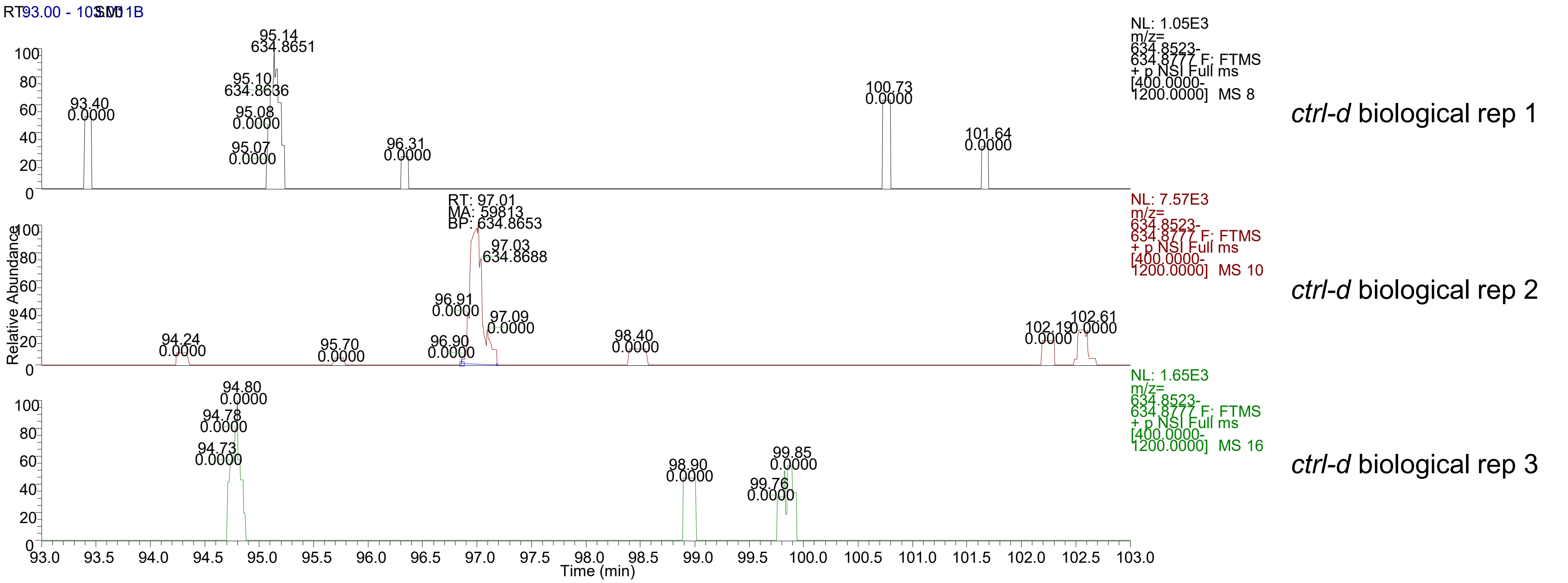

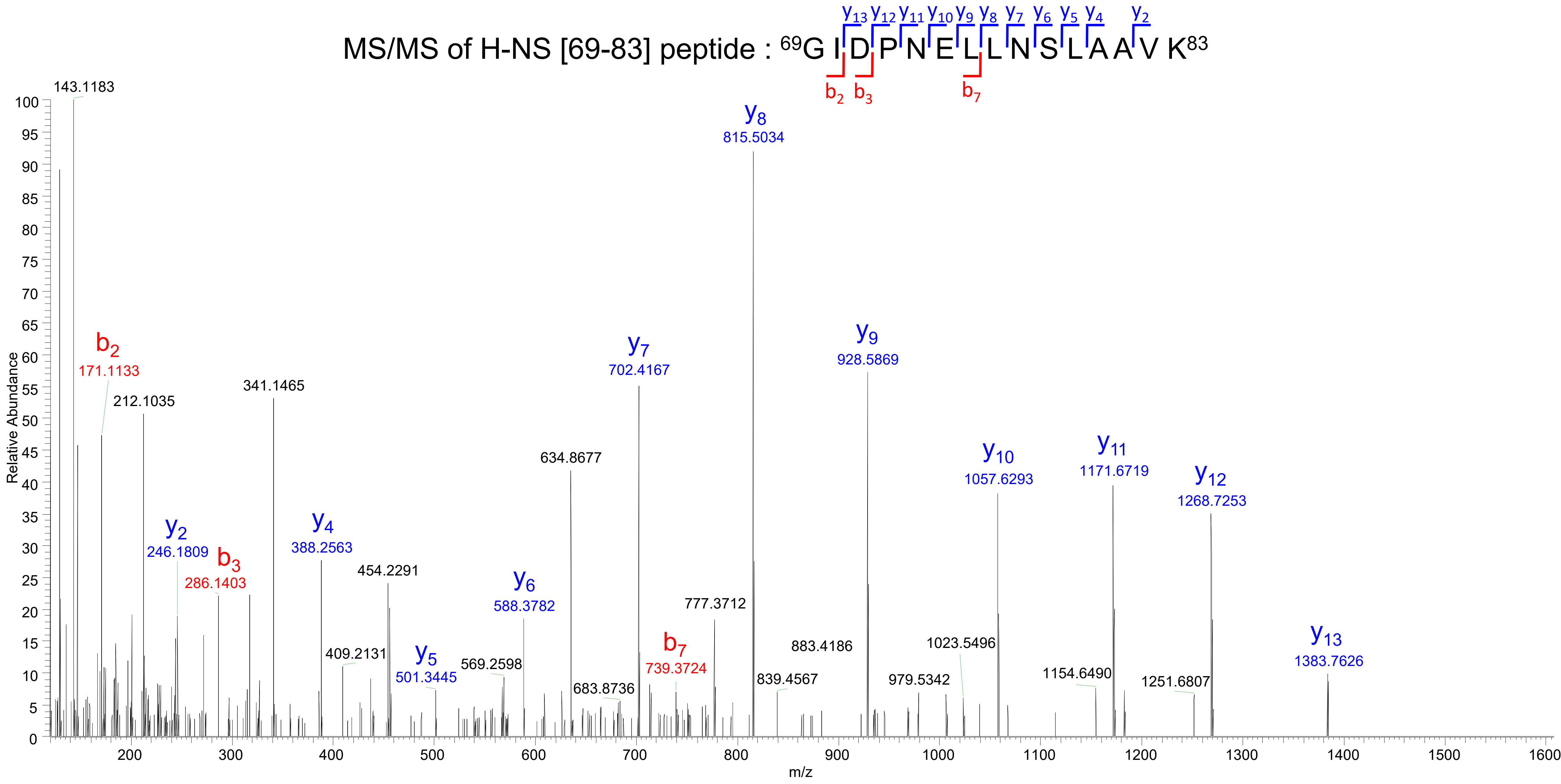
MS/MS of H-NS [69-83] peptide : 69G I D P N E L L N S L A A V K83
y13 y12 y11 y10 y9 y8 y7 y6 y5 y4 y2
b2 b3 b7
143.1183
b2 171.1133
212.1035
y2 246.1809
b3 286.1403
341.1465
y4 388.2563
409.2131
454.2291
y5 501.3445
569.2598
y6 588.3782
634.8677
683.8736
y7 702.4167
b7 739.3724
777.3712
y8 815.5034
839.4567
883.4186
y9 928.5869
979.5342
1023.5496
y10 1057.6293
1154.6490
y11 1171.6719
1251.6807
y12 1268.7253
y13 1383.7626
Relative Abundance
m/z

Extracted ion chromatography of HNS[69-83] peptide

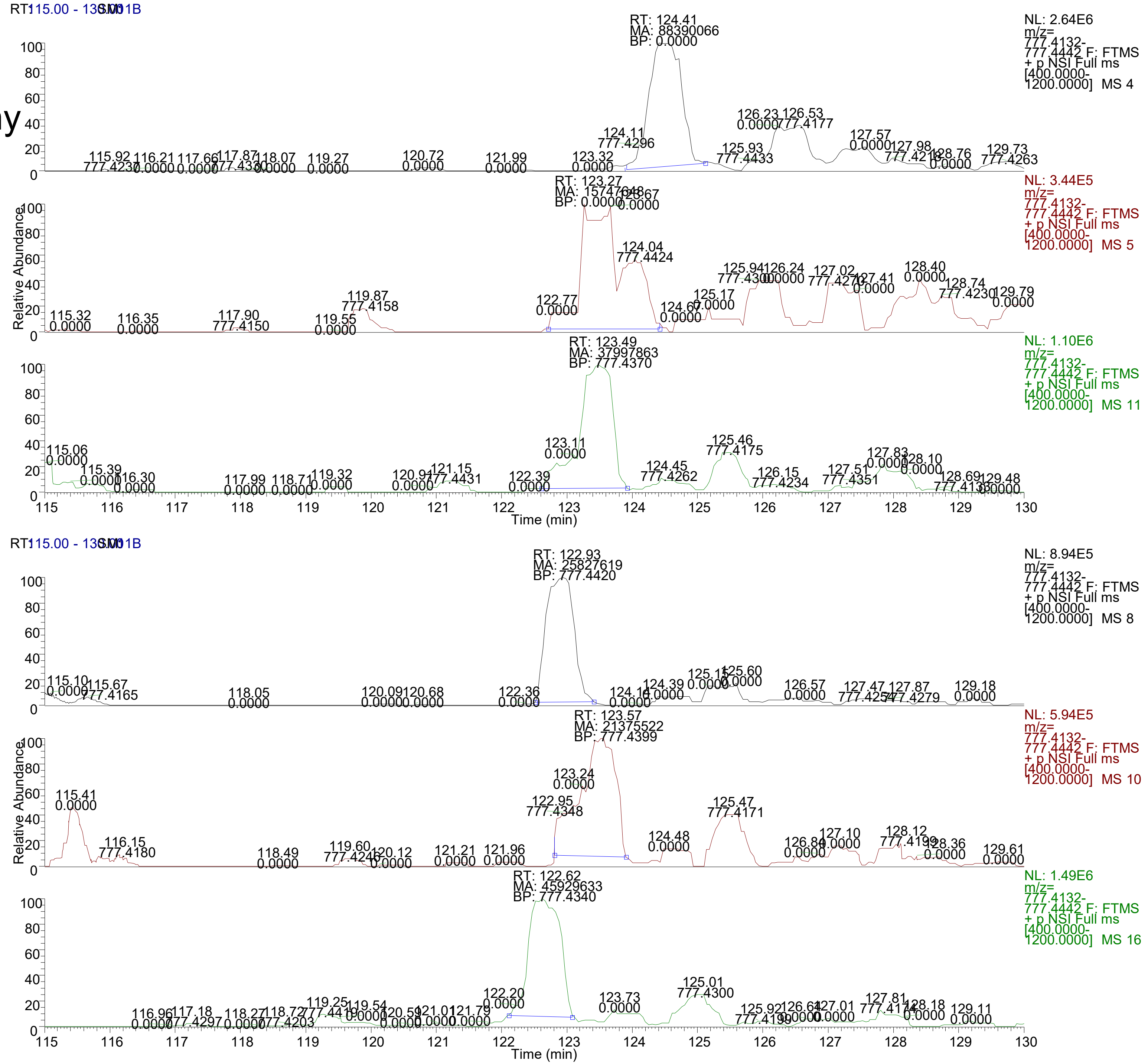

ispg-2d biological rep 1

ispg-2d biological rep 2

ispg-2d biological rep 3

ctrl-d biological rep 1

ctrl-d biological rep 2

ctrl-d biological rep 3
