# Supplementary material for: An evolutionarily conserved metabolite inhibits biofilm formation in Escherichia coli K-12
Source: Nat Commun. 2024 Nov 21;15:10079. doi: 10.1038/s41467-024-54501-w (PMC11582573; doi:10.1038/s41467-024-54501-w)
Supplement: Supplementary file 9 — Supplementary Data 7 [file 41467_2024_54501_MOESM9_ESM.pdf]

Result

Supplementary Data 7:

Quality control data and plots for the samples with limited proteolysis treatment

Property	Value
Peptide Level:	
spectra number	436,616
scans number	377,919
peptides number	49,424
sequences number	28,844
proteins number	2,117
protein groups number	1,741
decoy spectra number	711
decoy peptides number	494
decoy proteins number	439
decoy protein groups number	17
Cleavage:	
Specific	68.51 % (33,858/49,424)
C-term specific	28.77 % (14,219/49,424)
N-term specific	1.85 % (916/49,424)
Non specific	0.87 % (431/49,424)
Modifications:	
Oxidation[M]	12.17 % (6,013/49,424)
Carbamidomethyl[C]	8.93 % (4,416/49,424)
Deamidated[N]	5.75 % (2,843/49,424)
Gln->pyro-Glu[AnyN-termQ]	1.90 % (937/49,424)
Gln->Lys[Q]	1.71 % (846/49,424)
Carbamidomethyl[AnyN-term]	0.98 % (483/49,424)
Oxidation[Y]	0.76 % (377/49,424)
Trioxidation[W]	0.73 % (359/49,424)
Acetyl[AnyN-term]	0.54 % (268/49,424)
Oxidation[F](Phe->Tyr[F])	0.53 % (261/49,424)
Missed Cleavage:	
number=0	79.09 % (39,087/49,424)
number=1	18.07 % (8,933/49,424)
number=2	2.49 % (1,233/49,424)
number=3	0.29 % (145/49,424)
number=4	0.04 % (20/49,424)
number=5	0.00 % (2/49,424)
number=6	0.00 % (2/49,424)
number=7	0.00 % (1/49,424)
number=8	0.00 % (1/49,424)
Mixed Spectra:	
number=1	85.84 % (324,406/377,919)
number=2	12.88 % (48,662/377,919)
number=3	1.20 % (4,530/377,919)
number=4	0.08 % (309/377,919)
number=5	0.00 % (12/377,919)
Charge:	
charge=2	57.50 % (28,419/49,424)
charge=3	34.60 % (17,099/49,424)
charge=4	6.74 % (3,333/49,424)
charge=5	1.00 % (496/49,424)
charge=6	0.16 % (77/49,424)
MassError:	
Precusor mass error: (mean)	6.71ppm
Precusor mass error: (std)	±4.87ppm
ID Rate:	
10	18.96 % (15,704/82,820)
11	22.35 % (18,952/84,807)
13	26.73 % (24,965/93,384)
16	22.92 % (20,340/88,746)
19	49.79 % (64,728/130,000)
1_20230318155105	34.16 % (35,319/103,381)
2_20230318182707	36.78 % (38,654/105,091)
30	37.42 % (49,010/130,957)
34	34.57 % (46,702/135,104)
4	26.63 % (26,342/98,935)
5	23.76 % (23,922/100,690)
8	18.81 % (13,281/70,590)
Overall	30.86 % (377,919/1,224,505)

Graph

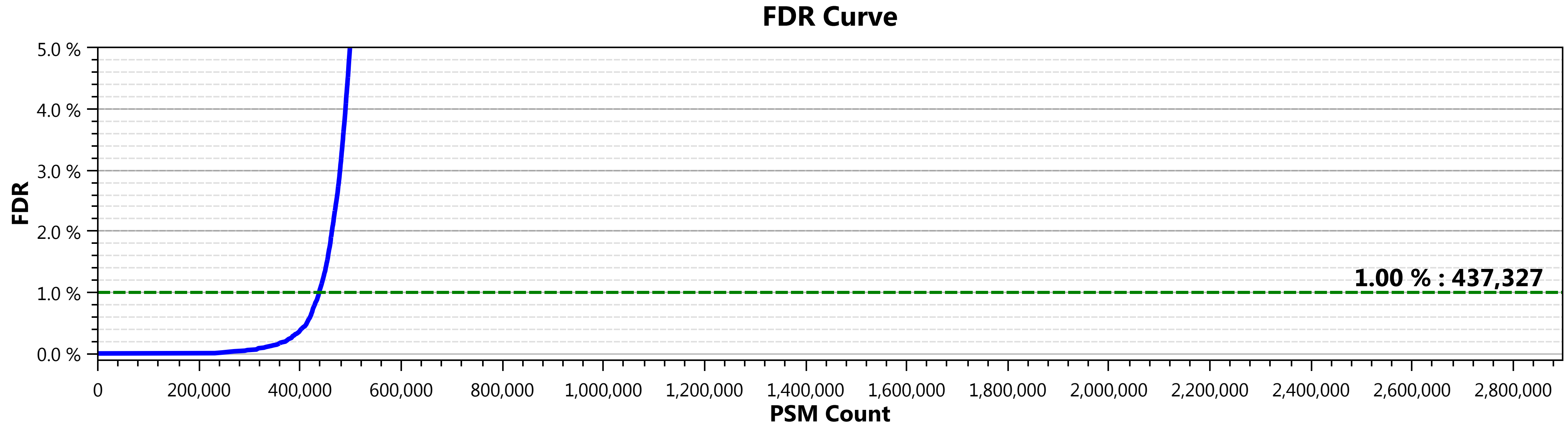
FDR Curve
FDR
5.0 %
4.0 %
3.0 %
2.0 %
1.0 %
0.0 %
1.00 % : 437,327
0
200,000
400,000
600,000
800,000
1,000,000
1,200,000
1,400,000
1,600,000
1,800,000
2,000,000
2,200,000
2,400,000
2,600,000
2,800,000
PSM Count

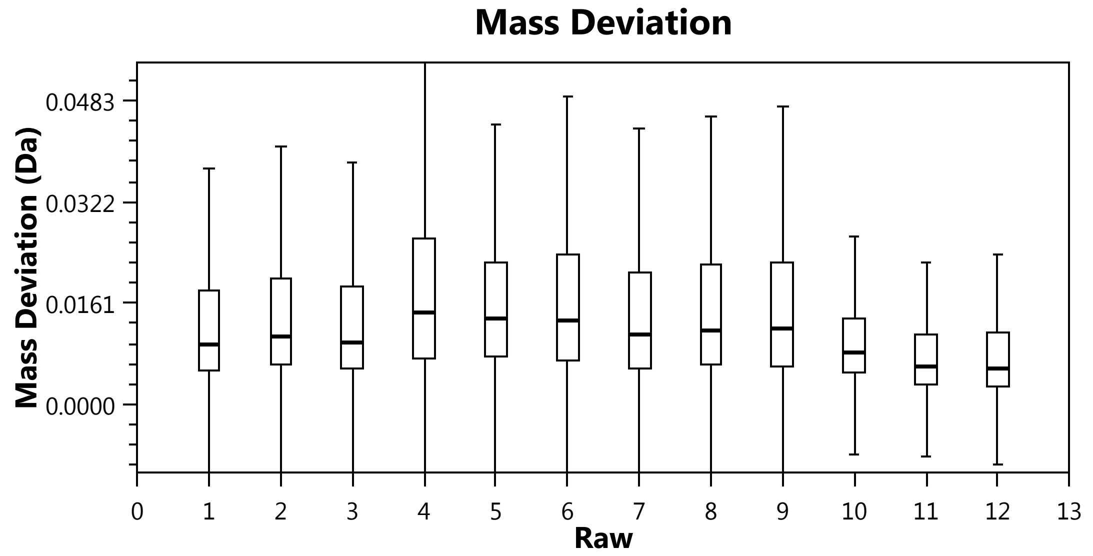
Mass Deviation
Mass Deviation (Da)
0.0483
0.0322
0.0161
0.0000
0 1 2 3 4 5 6 7 8 9 10 11 12 13
Raw

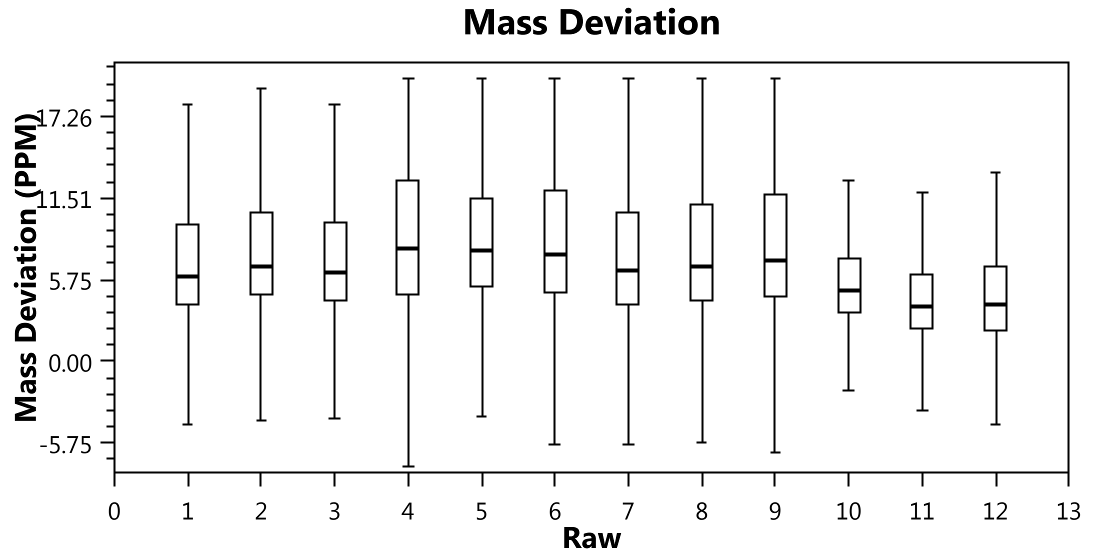
Mass Deviation
Mass Deviation (PPM)
17.26
11.51
5.75
0.00
-5.75
0 1 2 3 4 5 6 7 8 9 10 11 12 13
Raw

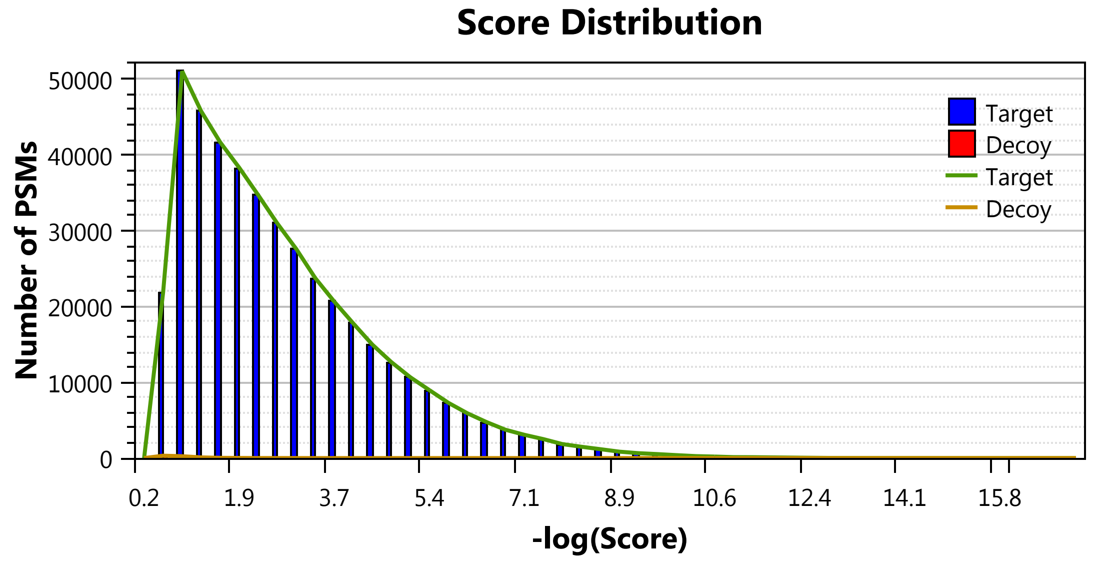
Score Distribution
Number of PSMs
50000
40000
30000
20000
10000
0
Target
Decoy
Target
Decoy
0.2 1.9 3.7 5.4 7.1 8.9 10.6 12.4 14.1 15.8
-log(Score)

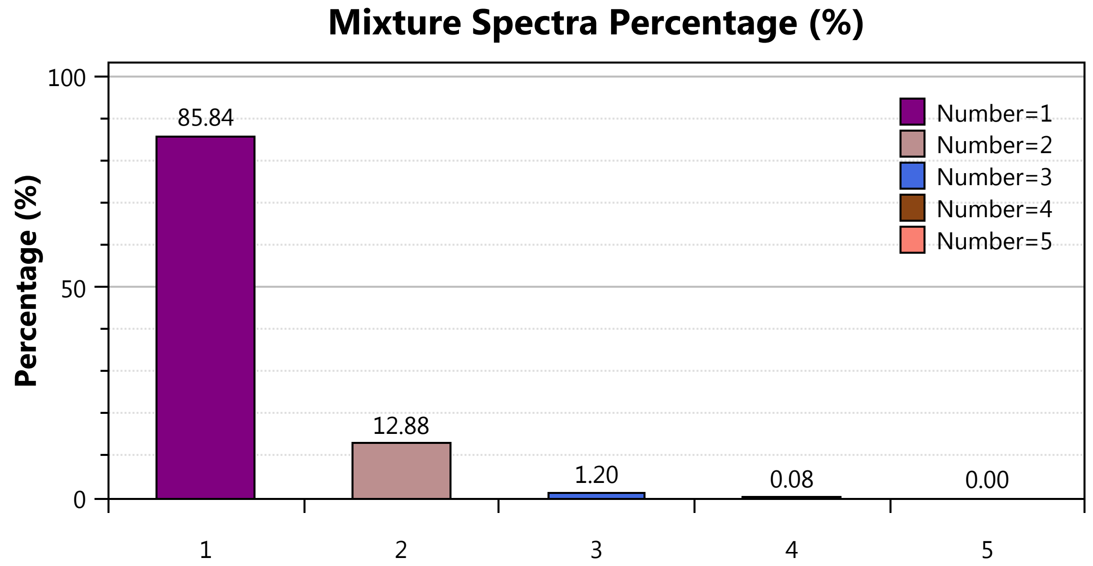
Mixture Spectra Percentage (%)
Percentage (%)
100
50
0
85.84
12.88
1.20
0.08
0.00
1 2 3 4 5
Number=1
Number=2
Number=3
Number=4
Number=5

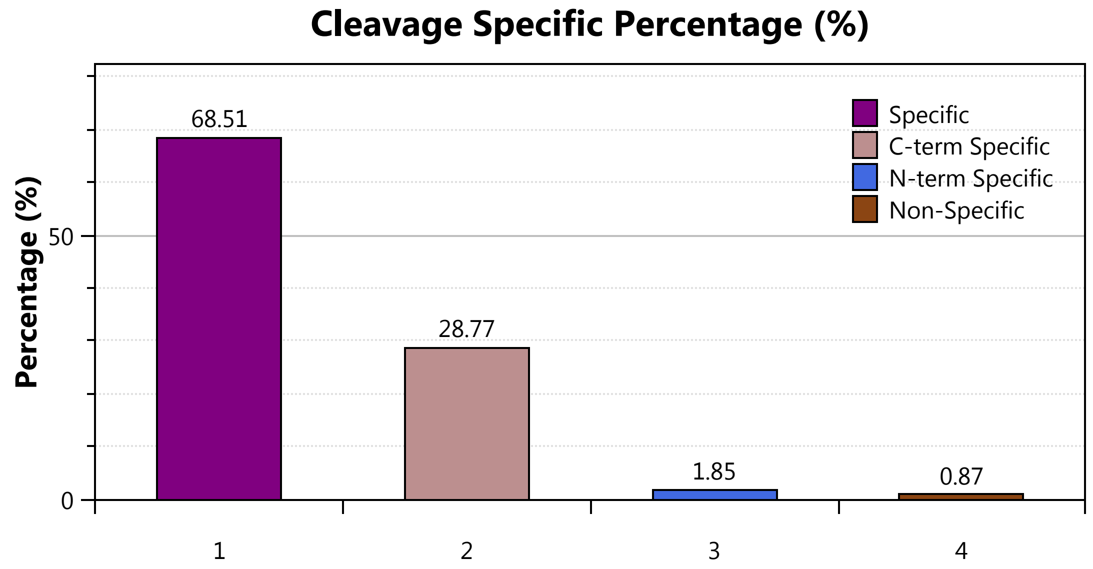
Cleavage Specific Percentage (%)
Percentage (%)
50
0
68.51
28.77
1.85
0.87
1 2 3 4
Specific
C-term Specific
N-term Specific
Non-Specific

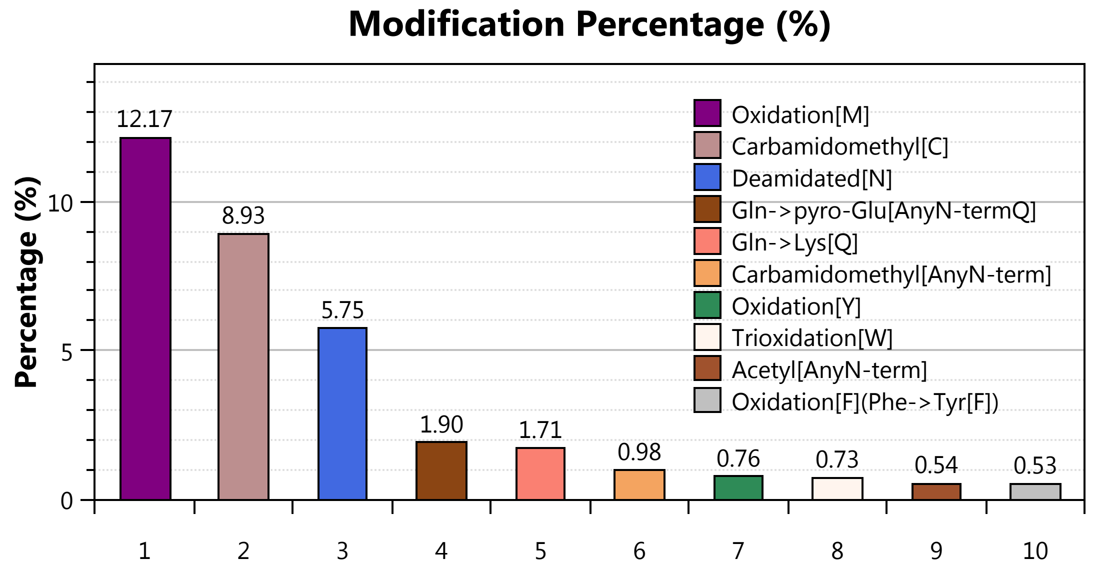
Modification Percentage (%)
Percentage (%)
10
5
0
12.17
8.93
5.75
1.90
1.71
0.98
0.76
0.73
0.54
0.53
1 2 3 4 5 6 7 8 9 10
Oxidation[M]
Carbamidomethyl[C]
Deamidated[N]
Gln->pyro-Glu[AnyN-termQ]
Gln->Lys[Q]
Carbamidomethyl[AnyN-term]
Oxidation[Y]
Trioxidation[W]
Acetyl[AnyN-term]
Oxidation[F](Phe->Tyr[F])

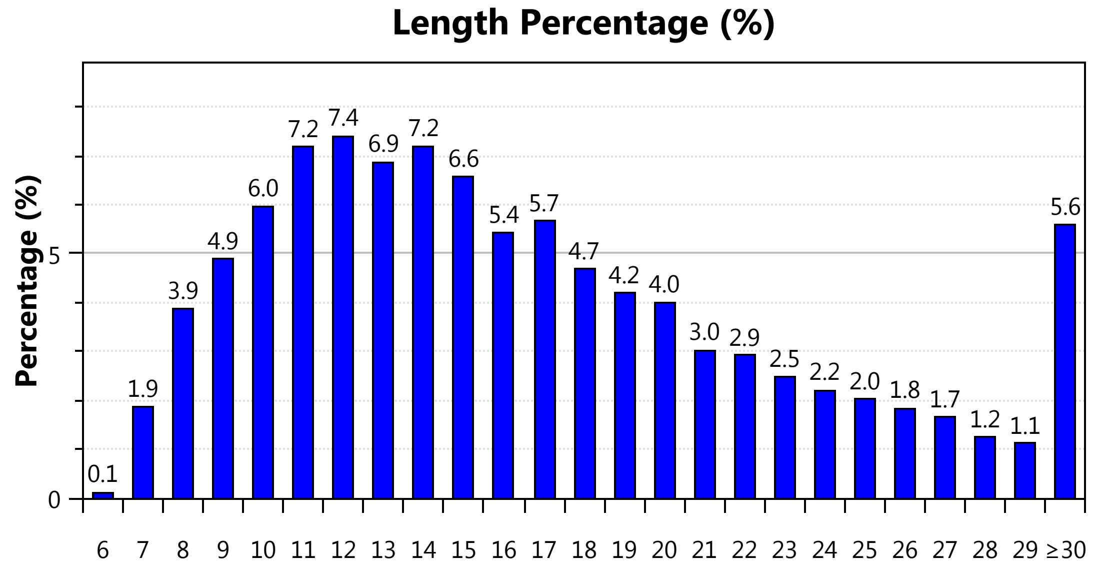
Length Percentage (%)
Percentage (%)
5
0
0.1 1.9 3.9 4.9 6.0 7.2 7.4 6.9 7.2 6.6 5.4 5.7 4.7 4.2 4.0 3.0 2.9 2.5 2.2 2.0 1.8 1.7 1.2 1.1 5.6
6 7 8 9 10 11 12 13 14 15 16 17 18 19 20 21 22 23 24 25 26 27 28 29 ≥30

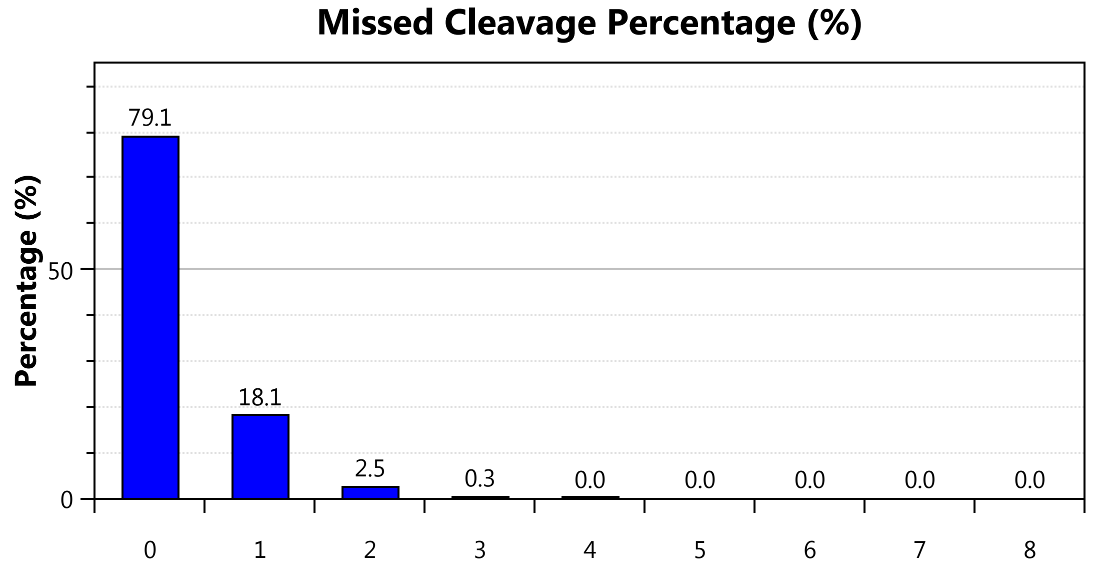
Missed Cleavage Percentage (%)
Percentage (%)
50
0
79.1
18.1
2.5
0.3
0.0
0.0
0.0
0.0
0.0
0 1 2 3 4 5 6 7 8

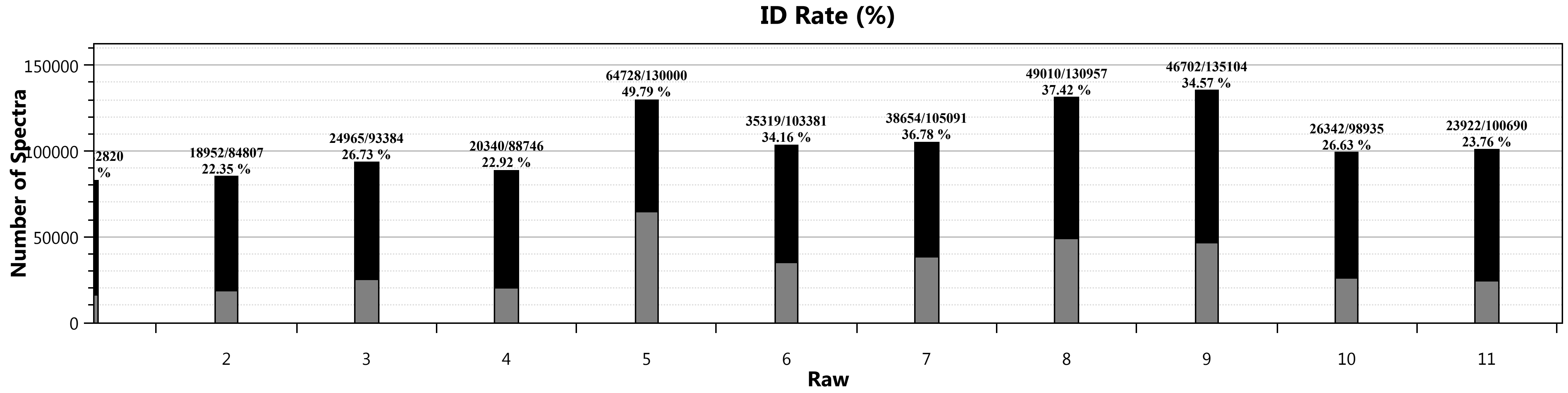
ID Rate (%)
Number of Spectra
150000
100000
50000
0
2820
%
18952/84807
22.35 %
24965/93384
26.73 %
20340/88746
22.92 %
64728/130000
49.79 %
35319/103381
34.16 %
38654/105091
36.78 %
49010/130957
37.42 %
46702/135104
34.57 %
26342/98935
26.63 %
23922/100690
23.76 %
2 3 4 5 6 7 8 9 10 11
Raw
