# Supplementary material for: An evolutionarily conserved metabolite inhibits biofilm formation in Escherichia coli K-12
Source: Nat Commun. 2024 Nov 21;15:10079. doi: 10.1038/s41467-024-54501-w (PMC11582573; doi:10.1038/s41467-024-54501-w)
Supplement: Supplementary file 10 — Reporting Summary [file 41467_2024_54501_MOESM10_ESM.pdf]

nature portfolio

Corresponding author(s): Katayoon Dehesh
Last updated by author(s): Nov 4, 2024

Reporting Summary

Nature Portfolio wishes to improve the reproducibility of the work that we publish. This form provides structure for consistency and transparency in reporting. For further information on Nature Portfolio policies, see our Editorial Policies and the Editorial Policy Checklist.

Statistics

For all statistical analyses, confirm that the following items are present in the figure legend, table legend, main text, or Methods section.

n/a	Confirmed	
☐	☒	The exact sample size (*n*) for each experimental group/condition, given as a discrete number and unit of measurement
☐	☒	A statement on whether measurements were taken from distinct samples or whether the same sample was measured repeatedly
☐	☒	The statistical test(s) used AND whether they are one- or two-sided<br>*Only common tests should be described solely by name; describe more complex techniques in the Methods section.*
☒	☐	A description of all covariates tested
☐	☒	A description of any assumptions or corrections, such as tests of normality and adjustment for multiple comparisons
☐	☒	A full description of the statistical parameters including central tendency (e.g. means) or other basic estimates (e.g. regression coefficient) AND variation (e.g. standard deviation) or associated estimates of uncertainty (e.g. confidence intervals)
☐	☒	For null hypothesis testing, the test statistic (e.g. *F*, *t*, *r*) with confidence intervals, effect sizes, degrees of freedom and *P* value noted<br>*Give P values as exact values whenever suitable.*
☒	☐	For Bayesian analysis, information on the choice of priors and Markov chain Monte Carlo settings
☒	☐	For hierarchical and complex designs, identification of the appropriate level for tests and full reporting of outcomes
☒	☐	Estimates of effect sizes (e.g. Cohen's *d*, Pearson's *r*), indicating how they were calculated

Our web collection on statistics for biologists contains articles on many of the points above.

Software and code

Policy information about availability of computer code

Data collection	Atomic force microscopy (AFM) data are collected using JPK NanoWizard /SPM software. SoftMax Pro 7 Software (Molecular Devices) is used for data collection from the Molecular Devices SpectraMax iD5 plate reader.
Data analysis	For targeted Metabolomics of the Central Carbon and Energy Metabolism (CCEM), peak integration was performed using the MultiQuant software version 3.0.3 (Sciex, Toronto, CA). RNA-seq data are analyzed using HISAT2 and DESeq2. ImageJ with FIJI is used for linear adjustment of images. QPCR data are analyzed using qBase+ (v3.4) software. Gwyddion 2.61 is used for reading and exporting the AFM raw data. Raw data of the LiP-MS were analyzed using the Proteome Discoverer (PD) (version 3.0, ThermoFisher scientific), Extracted ion chromatogram (XIC) peak areas and MS/MS peptide spectra were obtained using Xcalibur software (version 4.7, ThermoFisher scientific). LiP-MS results are analyzed using Microsoft Excel and GraphPad Prism. GraphPad Prism 8 software is used for Statistical analyses and generating plots. Figures are assembled in Microsoft PowerPoint. Line-arts in figures 3c and 5 are generated using using CorelDRAW Home & Student X8.

For manuscripts utilizing custom algorithms or software that are central to the research but not yet described in published literature, software must be made available to editors and reviewers. We strongly encourage code deposition in a community repository (e.g. GitHub). See the Nature Portfolio guidelines for submitting code & software for further information.

Data

Policy information about availability of data

All manuscripts must include a data availability statement. This statement should provide the following information, where applicable:

- Accession codes, unique identifiers, or web links for publicly available datasets
- A description of any restrictions on data availability
- For clinical datasets or third party data, please ensure that the statement adheres to our policy

Source data are provided with this paper.
The RNA-seq data have been deposited in the NCBI Sequence Read Archive (SRA) under accession number PRJNA1150654.
The raw mass spectrometry proteomics data of the LiP-MS analyses have been deposited to the MassIVE repository (https://massive.ucsd.edu) with the dataset identifier MSV000094552
The CCEM metabolomics data are available at MetaboLights repository under the accession number MTBLS2240.

Research involving human participants, their data, or biological material

Policy information about studies with human participants or human data. See also policy information about sex, gender (identity/presentation), and sexual orientation and race, ethnicity and racism.

Reporting on sex and gender	Not applicable
Reporting on race, ethnicity, or other socially relevant groupings	Not applicable
Population characteristics	Not applicable
Recruitment	Not applicable
Ethics oversight	Not applicable

Note that full information on the approval of the study protocol must also be provided in the manuscript.

Field-specific reporting

Please select the one below that is the best fit for your research. If you are not sure, read the appropriate sections before making your selection.

☒ Life sciences ☐ Behavioural & social sciences ☐ Ecological, evolutionary & environmental sciences

For a reference copy of the document with all sections, see nature.com/documents/nr-reporting-summary-flat.pdf

Life sciences study design

All studies must disclose on these points even when the disclosure is negative.

Sample size	At least three biological repeats for all quantitative experiments
Data exclusions	In the targeted metabolomic analyses focusing on central carbon and energy metabolism (Supplementary Data 1), we excluded sample ispg-2d-3 which shows much lower peak area than the other 4 biological replicates in the same group and was removed as an outlier.
Replication	We have at least 3 replications for most experiments except the LiP-MS and RNA-seq experiments, which are only conducted once but with three biological repeats.
Randomization	We randomized the samples for the LiP-MS assay and MEcPP measurement in E. coli cells of various genotypes and benzyl viologen treatment.
Blinding	Single-blind study is applied for LiP-MS assay including protein sample preparation, data acquisition and processing.

Reporting for specific materials, systems and methods

We require information from authors about some types of materials, experimental systems and methods used in many studies. Here, indicate whether each material, system or method listed is relevant to your study. If you are not sure if a list item applies to your research, read the appropriate section before selecting a response.

Materials & experimental systems

n/a	Involved in the study
☒	☐ Antibodies
☒	☐ Eukaryotic cell lines
☒	☐ Palaeontology and archaeology
☒	☐ Animals and other organisms
☒	☐ Clinical data
☒	☐ Dual use research of concern
☒	☐ Plants

Methods

n/a	Involved in the study
☒	☐ ChIP-seq
☒	☐ Flow cytometry
☒	☐ MRI-based neuroimaging

Plants

Seed stocks

Report on the source of all seed stocks or other plant material used. If applicable, state the seed stock centre and catalogue number. If plant specimens were collected from the field, describe the collection location, date and sampling procedures.

Novel plant genotypes

Describe the methods by which all novel plant genotypes were produced. This includes those generated by transgenic approaches, gene editing, chemical/radiation-based mutagenesis and hybridization. For transgenic lines, describe the transformation method, the number of independent lines analyzed and the generation upon which experiments were performed. For gene-edited lines, describe the editor used, the endogenous sequence targeted for editing, the targeting guide RNA sequence (if applicable) and how the editor was applied.

Authentication

Describe any authentication procedures for each seed stock used or novel genotype generated. Describe any experiments used to assess the effect of a mutation and, where applicable, how potential secondary effects (e.g. second site T-DNA insertions, mosiacism, off-target gene editing) were examined.
